# Graphene oxide polarizes iNKT cells for production of TGFβ and attenuates inflammation in an iNKT cell-mediated sepsis model

**DOI:** 10.1038/s41598-018-28396-9

**Published:** 2018-07-04

**Authors:** Sung Won Lee, Hyun Jung Park, Luc Van Kaer, Suklyun Hong, Seokmann Hong

**Affiliations:** 1 Department of Integrative Bioscience and Biotechnology, Institute of Anticancer Medicine Development, Sejong University, Seoul, 05006 Korea; 2 Graphene Research Institute, Sejong University, Seoul, 05006 Korea; 3 Department of Pathology, Microbiology and Immunology, Vanderbilt University School of Medicine, Nashville, TN 37232 USA; 4 Department of Physics, Sejong University, Seoul, 05006 Korea

## Abstract

Graphene oxide (GO) modulates the functions of antigen-presenting cells including dendritic cells (DCs). Although carbon nanotubes affect expression of the MHC class I-like CD1d molecule, whether GO can influence immune responses of CD1d-dependent invariant natural killer T (iNKT) cells remains unclear. Here, we investigated the impact of GO on inflammatory responses mediated by α-galactosylceramide (α-GalCer), an iNKT cell agonist. We found that *in vivo* GO treatment substantially inhibited the capacity of α-GalCer to induce the iNKT cell-mediated trans-activation of and cytokine production by innate and innate-like cells, including DCs, macrophages, NK cells, and γδ T cells. Such effects of GO on α-GalCer-induced inflammatory responses closely correlated with iNKT cell polarization towards TGFβ production, which also explains the capacity of GO to expand regulatory T cells. Interestingly, the absence of TLR4, a receptor for GO, failed to downregulate, and instead partially enhanced the anti-inflammatory activity of GO against α-GalCer-elicited responses, implying negative effects of TLR4 signaling on the anti-inflammatory properties of GO. By employing an α-GalCer-induced sepsis model, we further demonstrated that GO treatment significantly protected mice from α-GalCer-induced lethality. Taken together, we provide strong evidence that GO holds promise as an adjuvant to modulate iNKT cell responses for immunotherapy.

## Introduction

Sepsis, known as a systemic inflammatory response syndrome (SIRS), is a life-threatening illness triggered by the systemic release of microbial products during infection. Tissue damage during sepsis is caused by a “cytokine storm,” resulting from high levels of pro-inflammatory cytokines such as IL6 and TNFα^1^. Diverse microbial products can precipitate the development of sepsis. For example, lipopolysaccharide (LPS) derived from Gram-negative bacteria, may cause sepsis by engaging Toll-like receptor 4 (TLR4) on phagocytes and other cell types. Additionally, glycolipids from *Streptococcus pneumonia*, which is responsible for high mortality in >65-year-old people, can induce septic shock by activating invariant natural killer T (iNKT) cells^2^.

iNKT cells are a group of innate-like T lymphocytes that recognize glycolipid antigens presented onto CD1d, an MHC class I-like protein. Upon antigen stimulation, iNKT cells rapidly produce either pro-inflammatory (TNFα, IFNγ, and IL17) or anti-inflammatory (IL4 and IL10) cytokines, or both^3^. Upon stimulation with the iNKT cell agonist α-Galactosylceramide (α-GalCer), iNKT cells interact with various innate and innate-like immune cells (e.g., dendritic cells (DCs), NK cells, and γδ T cells), leading to enhanced inflammatory responses^4,5^. Due to the potent adjuvant effects of α-GalCer on innate immunity, α-GalCer-activated iNKT cells can potently modulate adaptive immune responses of both CD4^+^ and CD8^+^ T cells^6^. Activation of iNKT cells in this manner may promote Th1-mediated immune responses that induce tissue injury. For example, IFNγ-producing iNKT cells enhance the pathogenesis of sepsis by inducing secretion of complement protein C5a^7^. Moreover, stimulation of iNKT cells in response to co-administration of either α-GalCer plus D-galactosamine (D-GalN)^8^ or α-GalCer plus LPS^9^ in mice induces liver injury through IFNγ and TNFα production, ultimately resulting in increased death as compared with α-GalCer or LPS treatment alone. Mechanistically, iNKT cell-mediated liver injury was mediated through up-regulation of hepatocyte-derived IRF-1 in response to IFNγ and TNFα^10^.

Graphene oxide (GO) is a single-atomic layered material composed of carbon, which is produced by the oxidation and exfoliation of graphite, and shows excellent physical properties, such as high strength and superior electron mobility. GO has a variety of biomedical applications, ranging from gene delivery to stem cell differentiation and cancer therapy. The potential therapeutic applications of GO in patients have prompted investigations of its effects on the immune system. The majority of studies on graphene-family nanomaterials have focused on cytotoxic effects against immune cells^11^. These studies revealed GO-mediated necrotic cell death of macrophages via TLR4 signaling and autocrine TNFα production^12^. Moreover, GO can modulate immune functions. Administration of GO suppressed antigen-specific Th2 responses in an ovalbumin (OVA)-induced asthma model, resulting in attenuation of airway inflammation^13^. GO treatment inhibits the presentation of antigenic peptides through down-regulation of immunoproteasomes, which are required for processing of MHC class I-presented antigens in DCs^14^. However, the effects of GO on the presentation of glycolipid antigens by CD1d and the responses mediated by CD1d-restricted iNKT cells remain unexplored.

Here, we report that GO treatment significantly inhibits pro-inflammatory cytokine production in response to iNKT cell activation by α-GalCer, which involved modulation of both CD1d-mediated antigen presentation and iNKT cell responses. Consequently, GO also suppressed innate immune responses elicited by α-GalCer-mediated iNKT cell activation. The immunomodulatory effects of GO on α-GalCer-mediated immune responses involved polarization of iNKT cell responses from IL4/IFNγ production towards TGFβ production, which subsequently led to an expansion of regulatory T (Treg) cells. Moreover, *in vivo* GO treatment attenuated lethality induced by α-GalCer. These findings highlight GO as an excellent drug delivery carrier for treatment of septic shock.

## Results

### GO suppresses innate immune responses elicited by α-GalCer-stimulated iNKT cells

We investigated whether GO can modulate immune responses using a model of iNKT cell activation with α-GalCer. The characteristics of GO used in this study are described in supplementary figures. The major elements of GO were determined using elemental analyzers (Supplementary Fig. 1a,b) and Raman spectra were generated to obtain structural information on GO (Supplementary Fig. 1c). Analysis of morphology was performed by TEM (Supplementary Fig. 1d). As expected, we found that GO used in the experiments showed general properties of GO reported in previous publications^15–17^.

**Figure 1:**
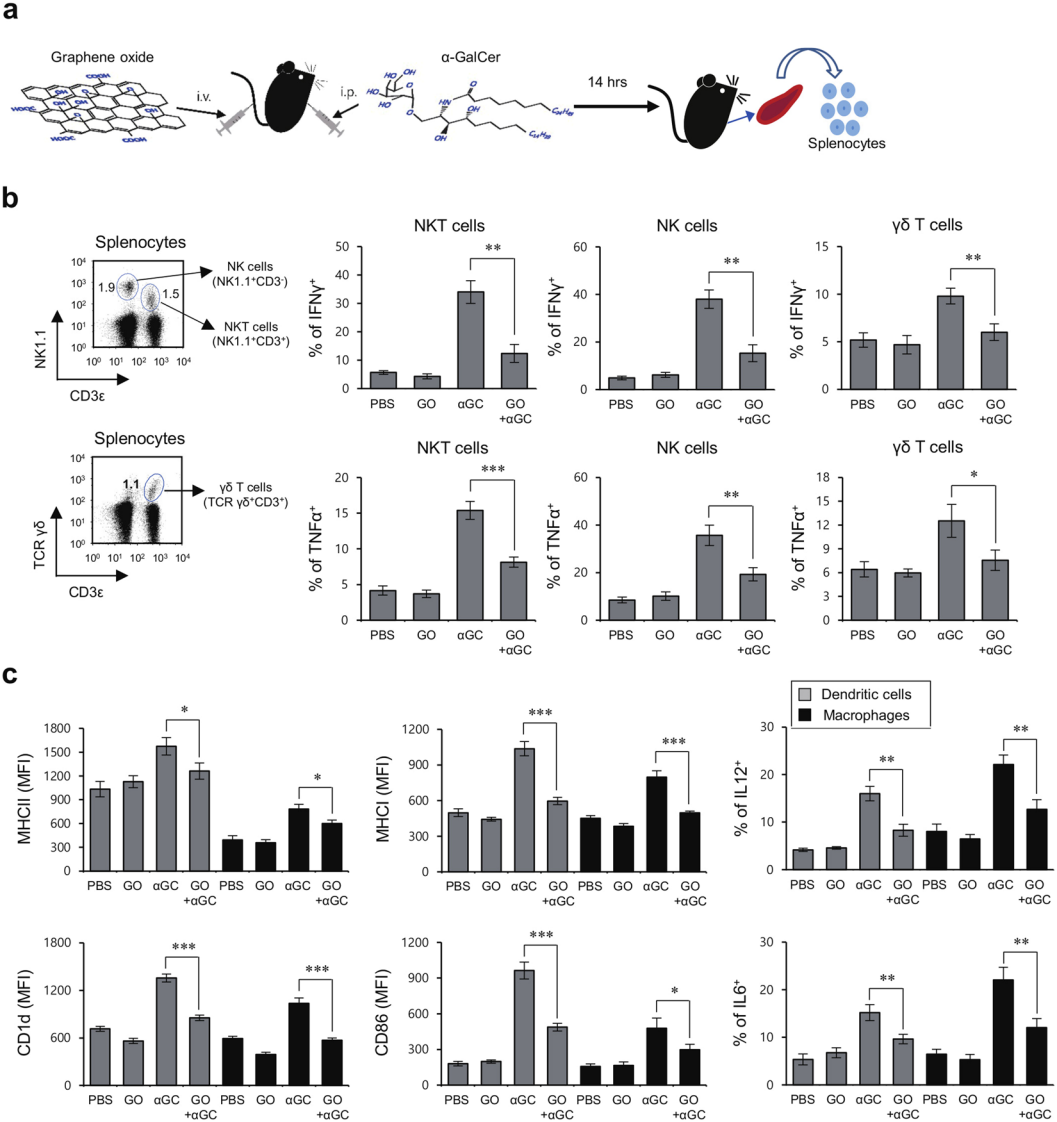
GO injection suppresses innate immune responses elicited by α-GalCer-stimulated iNKT cells. (**a**) Experimental scheme to examine the effect of GO in α-GalCer-induced immune responses. (**b,c**) WT B6 mice were i.p. injected with α-GalCer (2 μg) or PBS control and were concurrently i.v. injected with either GO (50 μg) or PBS control. Splenocytes were prepared from each experimental group at 14 hrs after treatment. (**b**) Total splenocytes were isolated from B6 mice. The frequencies of iNKT, NK, and γδ T cells were measured by gating on NK1.1^+^CD3ε^+^, NK1.1^+^CD3ε^−^, and TCRγδ^+^CD3ε^+^ populations, respectively (left panels). Intracellular IFNγ and TNFα production by iNKT, NK, and γδ T cells was determined by flow cytometry (right panels). The mean values ± SD are shown (unpaired two-tailed Student’s t-test; *P < 0.05, **P < 0.01, ***P < 0.001; n = 4 per group in the experiment). (**c**) Surface expression of MHC II, MHC I, CD86, and CD1d molecules as well as intracellular expression of IL12 and IL6 were analyzed in DCs and macrophages. The mean values ± SD are shown (unpaired two-tailed Student’s t-test; *P < 0.05, **P < 0.01, ***P < 0.001; n = 4 per group in the experiment).

Previously, it has been demonstrated that *in vivo* α-GalCer-activated iNKT cells potently trans-activate NK cells^4^ and also increase IFNγ production by innate-like γδ T cells, which promotes α-GalCer-enhanced Th1 immunity via an IL12-dependent mechanism^5^. To investigate how GO might affect α-GalCer-mediated activation of iNKT cells, NK cells, and γδ T cells, we injected GO i.v. into α-GalCer-treated mice, and assessed the subsequent production of IFNγ and TNFα by iNKT cells, NK cells, and γδ T cells (Fig. 1a). We observed that GO inhibited cytokine production by α-GalCer-activated iNKT cells, NK cells, and γδ T cells (Fig. 1b). Taken together, these data suggest that GO can block inflammatory responses initiated by the activation of CD1d-restricted iNKT cells.

iNKT cell-derived cytokines such as IFNγ enhance the maturation and cytokine production capacity of APCs, including DCs and macrophages. Thus, we analyzed the effect of GO on the capacity of α-GalCer to enhance APC functions *in vivo* by examining the surface expression of MHC I, MHC II, CD86, and CD1d, and secretion of IL12 and IL6 by DCs and macrophages following i.p. injection of α-GalCer. We found that α-GalCer upregulated surface expression of the co-stimulatory molecule CD86 and several antigen-presenting molecules (MHC I, MHC II, and CD1d). However, this upregulation was significantly attenuated upon GO injection. Moreover, while α-GalCer potently enhanced production of pro-inflammatory cytokines (IL12 and IL6) by APCs, this was blunted following i.v. injection of GO (Fig. 1c). Collectively, these results strongly indicate that GO can suppress α-GalCer-triggered iNKT cell activation in an APC-dependent manner.

### TLR4 signaling is not involved in the suppressive effects of GO on α-GalCer-mediated immune responses

**Figure 2:**
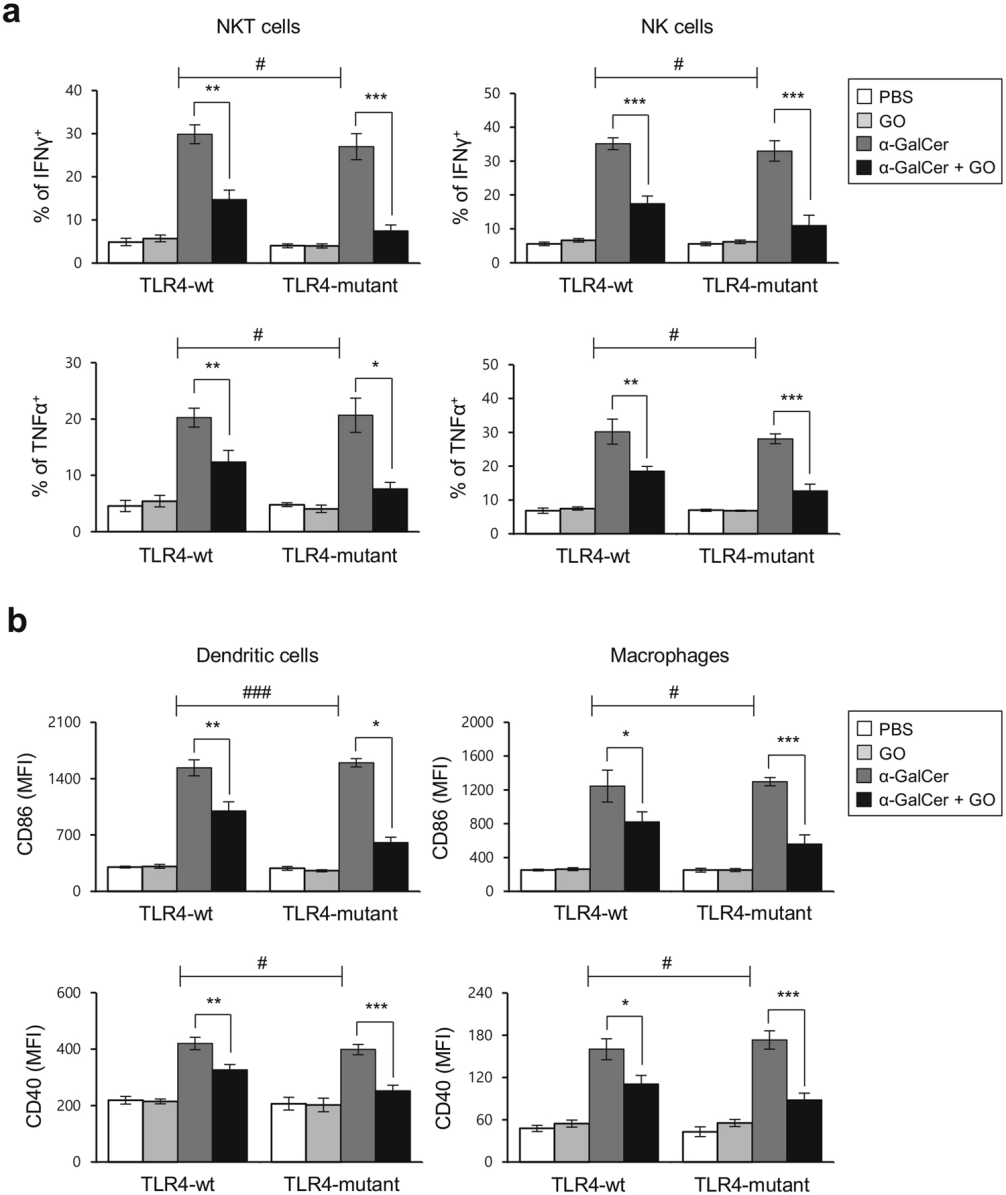
TLR4 signaling negatively impacts the suppressive effects of GO on α-GalCer-mediated immune responses. (**a**,**b**) C3H/HeN and C3H/HeJ mice were i.p. injected with α-GalCer (2 μg) or PBS control and were concurrently i.v. injected with either GO (50 μg) or PBS control. Fourteen hrs later, splenocytes were prepared from each experimental group. (**a**) Intracellular IFNγ (upper panels) and TNFα (lower panels) production in iNKT and NK cells was assessed by flow cytometry. The mean values ± SD are shown (unpaired two-tailed Student’s t-test; *P < 0.05, **P < 0.01, ***P < 0.001; n = 4 per group in the experiment). Two-way ANOVA (genotype × treatment) showed a significant difference between these two factors (^#^P < 0.05). (**b**) Expression of CD86 (upper panels) and CD40 (lower panels) in DCs and macrophages were assessed by flow cytometry. The mean values ± SD are shown (unpaired two-tailed Student’s t-test; *P < 0.05, **P < 0.01, ***P < 0.001; n = 4 per group in the experiment). Two-way ANOVA (genotype × treatment) showed a significant difference between these two factors (^#^P < 0.05 and ^###^P < 0.001).

It has been reported that activation of TLR4 signaling by GO induces necrosis of macrophages^12^, and that TLR4 engagement directly triggers iNKT cells to produce large amounts of IFNγ and IL4^18^. Thus, to determine whether TLR4 signaling mediates effects of GO on α-GalCer-mediated immune responses, we injected GO into either TLR4-mutant C3H/HeJ or TLR4-sufficient C3H/HeN mice and determined intracellular IFNγ and TNFα production by iNKT and NK cells by flow cytometry. Surprisingly, cells from both groups of GO-treated mice produced similar levels of IFNγ and TNFα, indicating that TLR4 signaling is not critical for modulating the effects of GO on α-GalCer-mediated immune responses (Fig. 2a).

We further investigated whether TLR4 expression influences the inhibitory activity of GO on the maturation of DCs and macrophages. To test this parameter, we examined CD86 and MHC II surface expression on DCs and macrophages from both C3H/HeJ and C3H/HeN mice following *in vivo* GO and α-GalCer treatment. In contrast to our hypothesis, we found that TLR4-deficiency partially enhanced the capacity of GO to inhibit APC maturation (Fig. 2b), indicating that GO interaction with TLR4 might interfere with the effects of GO on immune suppressive signaling pathways.

### GO attenuates α-GalCer-mediated septic shock by inducing anti-inflammatory innate immune responses in the liver

**Figure 3:**
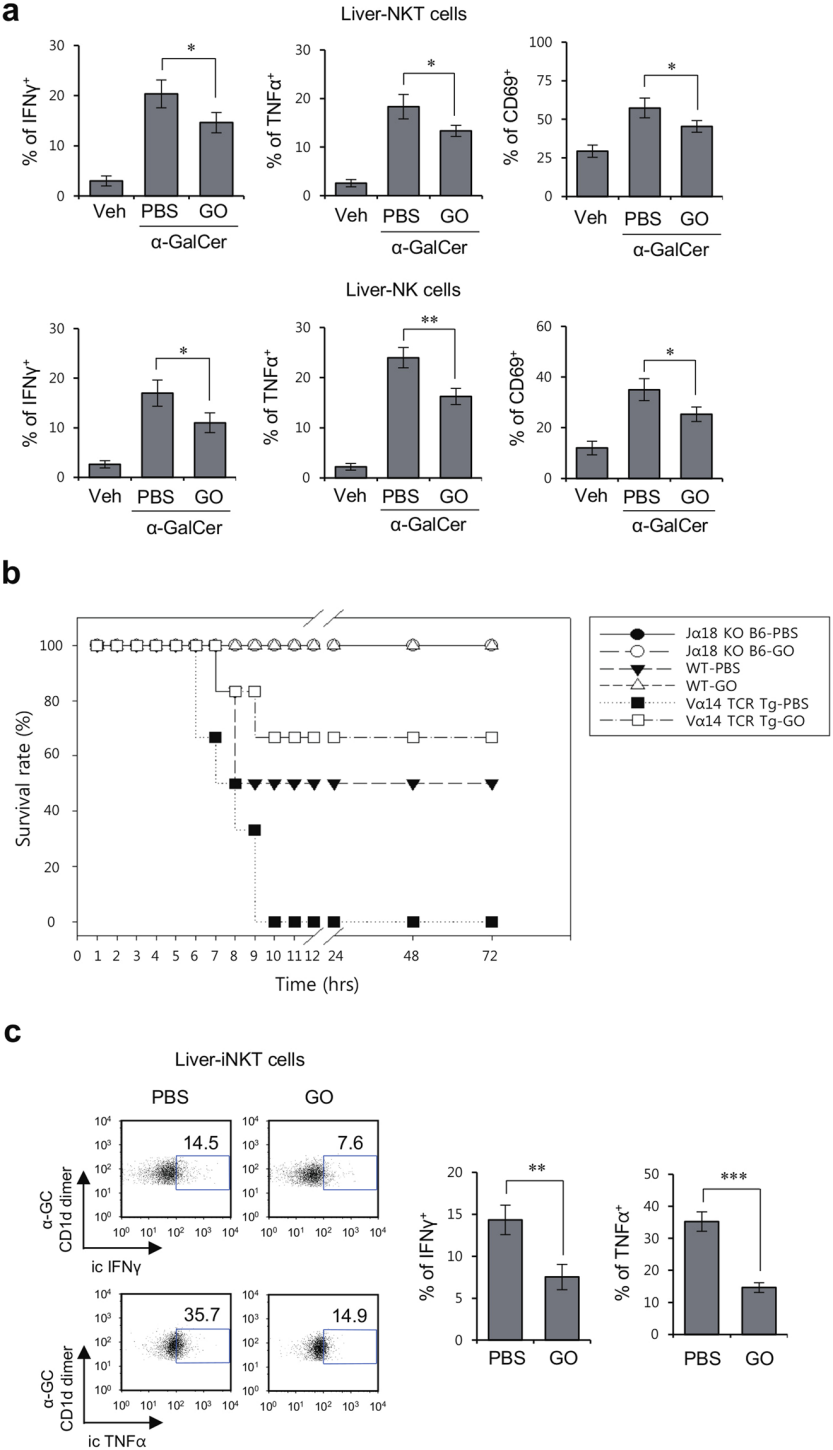
*In vivo* GO treatment protects mice against α-GalCer/D-GalN-induced septic shock. (**a**) WT B6 mice were i.p. injected with α-GalCer (2 μg) or PBS control and were concurrently i.v. injected with either GO (50 μg) or PBS control. Subsequently, hepatic leukocytes were prepared from each experimental group at 14 hrs after treatment. Cytokine production (IFNγ and TNFα) and CD69 expression in hepatic iNKT cells and NK cells from the liver of each experimental group were analyzed by flow cytometry. The mean values ± SD are shown (unpaired two-tailed Student’s t-test; *P < 0.05, **P < 0.01; n = 4 per group in the experiment). (**b,c**) WT, Jα18 KO, and Vα14 TCR Tg B6 mice were injected with either PBS or GO (50 μg) following i.v. injection of α-GalCer (2 μg/mouse)/D-GalN (10 mg/mouse). (**b**) The survival of these mice was monitored until 72 hrs after α-GalCer/D-GalN challenge. (n = 4 in Jα18 KO-PBS or GO; n = 6 in WT-PBS or GO; n = 6 in Vα14 TCR Tg-PBS or GO in the experiment) (**c**) IFNγ and TNFα production in liver iNKT cells of each experimental group were measured by flow cytometry. Representative data (left panels) and a summary (right panels) are shown. The mean values ± SD are shown (unpaired two-tailed Student’s t-test; **P < 0.01, ***P < 0.001; n = 3 in WT-PBS; n = 4 in WT-GO in the experiment).

The crosstalk between innate immune cells such as DCs, macrophages, NK cells, and iNKT cells can induce a cytokine storm during septic shock, consequently resulting in tissue injury and lethality^19^. In general, NK and iNKT cells accelerate liver injury by secreting pro-inflammatory cytokines such as IFNγ and TNFα^20^. Since our findings showed that GO suppresses inflammatory responses elicited by glycolipid-activated splenic iNKT and NK cells, we evaluated whether *in vivo* GO treatment can protect mice against α-GalCer-induced inflammatory responses in the liver. To address this issue, we analyzed cytokine production (IFNγ and TNFα) and CD69 expression in α-GalCer- and GO-treated WT B6 mice at 14 hrs after challenge. Hepatic iNKT and NK cells from GO-injected mice displayed reduced cytokine production and CD69 expression compared with PBS-injected mice (Fig. 3a).

Next, we examined whether *in vivo* GO treatment can modulate the development of lethal shock induced by activated iNKT cells. To test this possibility, we employed an α-GalCer/D-GalN-induced sepsis model and took advantage of iNKT cell-deficient (Jα18 KO) and iNKT cell-overexpressing (Vα14 TCR Tg) mice. GO was i.v. injected to WT, Jα18 KO, and Vα14 TCR Tg B6 mice following α-GalCer/D-GalN stimulation and the survival rate was assessed over a 72-hr time period. As expected, both WT and Vα14 TCR Tg B6 mice treated with GO + α-GalCer/D-GalN exhibited significantly reduced mortality rates (WT: 0% and Vα14 TCR Tg: 33.3%) compared with mice treated with α-GalCer/D-GalN alone (WT: 50% and Vα14 TCR Tg: 100%) (Fig. 3b). The survival rate of Jα18 KO B6 mice treated with either α-GalCer/D-GalN or GO + α-GalCer/D-GalN was 100% (Fig. 3b). Moreover, production of IFNγ and TNFα by hepatic iNKT cells was significantly reduced in WT B6 mice treated with GO + α-GalCer/D-GalN, compared with WT B6 mice treated with α-GalCer/D-GalN alone (Fig. 3c). These data clearly demonstrated that the GO-mediated functional alterations of iNKT cells contributed to the reduction of mortality in severe septic shock.

### GO induces a regulatory phenotype in iNKT cells

**Figure 4:**
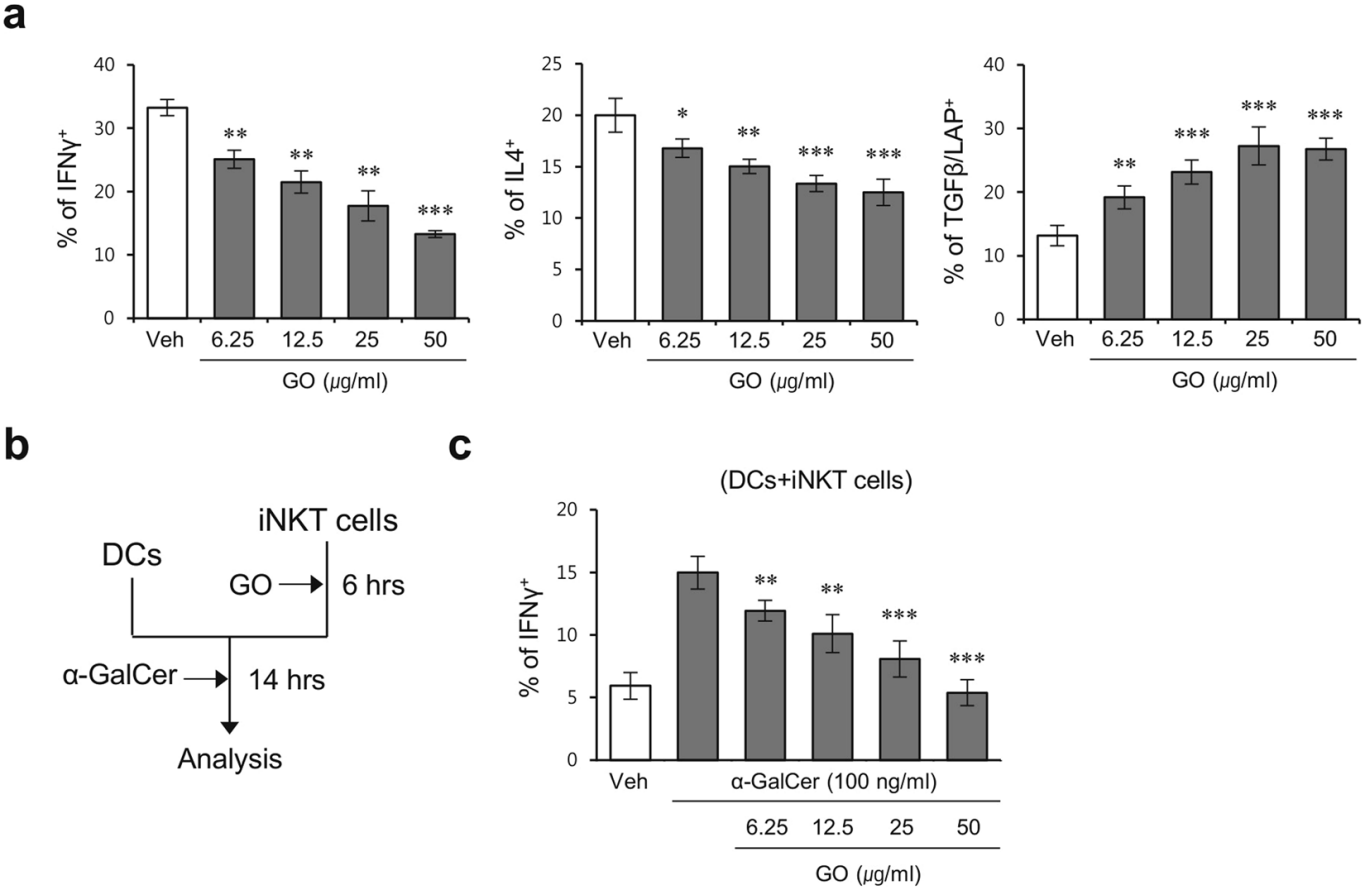
GO induces the conversion of iNKT cells into cells with a regulatory phenotype. (**a**) iNKT cells purified from B6 mice were cultured with GO (6.25, 12.5, 25, and 50 μg/ml) for 10 hrs and subsequently stimulated with PMA/ionomycin for 2 hrs. The cells were collected and intracellular IFNγ, IL4, and TGFβ production in iNKT cells was determined by flow cytometry. The mean values ± SD are shown (unpaired two-tailed Student’s t-test; *P < 0.05, **P < 0.01, ***P < 0.001; n = 4 per group in the experiment). (**b**) Experimental scheme to examine the effect of GO-stimulated iNKT cells in α-GalCer-induced immune responses. (**c**) iNKT cells purified from B6 mice were stimulated with GO and 6 hrs later these cells were separated from the remaining GO by density-gradient centrifugation using Lympholyte-M. DCs purified from Jα18 KO mice were cultured for 14 hrs with GO-stimulated iNKT cells in the presence of either α-GalCer or vehicle. The cells were collected and intracellular IFNγ production in iNKT cells was determined by flow cytometry. The mean values ± SD are shown (unpaired two-tailed Student’s t-test; **P < 0.01, ***P < 0.001; n = 3 per group in the experiment).

iNKT cells can secrete Th1 type (IFNγ) and Th2 type (IL4) cytokines during steady state conditions^21^. TGFβ signaling in iNKT cells decreases the production of effector cytokines such as IFNγ and IL4^22^, but increases their acquisition of a regulatory phenotype characterized by expression of CTLA4 and GITR^22,23^. Thus, to investigate whether GO can influence the phenotypic and cytokine production characteristics of iNKT cells, we purified iNKT cells using a MACS system, then treated these cells with GO *in vitro* in the absence of APCs for 10 hrs, and subsequently measured intracellular IFNγ, IL4, and TGFβ levels in iNKT cells by flow cytometry. We found that GO stimulation decreased IFNγ and IL4 secretion but increased TGFβ production by iNKT cells, implying that GO can contribute to the conversion of iNKT cells toward a regulatory phenotype (Fig. 4a).

Next, to test whether GO can directly affect iNKT cells in an APC-independent manner, we purified and stimulated iNKT cells with GO for 6 hrs and subsequently thoroughly washed these cells with PBS to remove any remaining GO. Next, we cultured these iNKT cells further with DCs plus α-GalCer, and 14 hrs later, we measured IFNγ production by iNKT cells using flow cytometry (Fig. 4b). We found that iNKT cells pre-treated with GO produced dramatically reduced levels of IFNγ as compared with cells pre-treated with PBS (Fig. 4c). These data clearly demonstrated that GO can modulate the phenotype of iNKT cells in a cell-intrinsic manner, in addition to being able to modulate these cells via APCs.

### GO induces the expansion of Treg cells in a manner that involves iNKT cells

**Figure 5:**
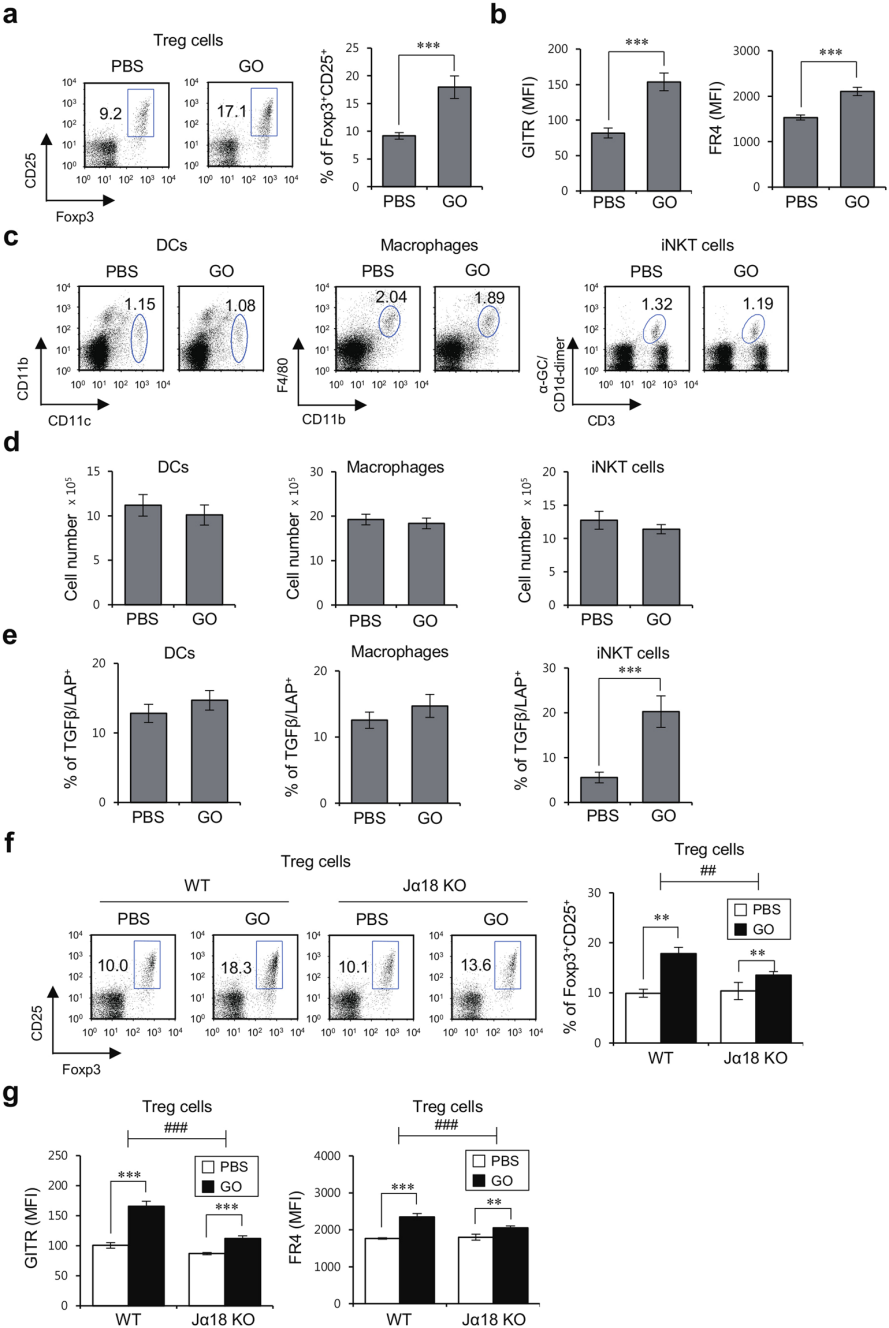
GO-mediated phenotypic changes in iNKT cells contribute to Foxp3^+^ Treg cell expansion. (**a**,**b**) WT B6 mice were i.v. injected with either PBS or GO (50 μg). Five days later, splenocytes were prepared from each group of mice. (**a**) The frequency of Foxp3^+^CD25^+^ Treg cells among total splenic CD4^+^ T cells from each experimental group was measured by flow cytometry. Representative data (left panels) and a summary (right panel) are shown. The mean values ± SD are shown (unpaired two-tailed Student’s t-test; ***P < 0.001; n = 4 per group in the experiment). (**b**) Expression of GITR and FR4 in CD4^+^CD25^+^Foxp3^+^ Treg cells was analyzed by flow cytometry. The mean values ± SD are shown (unpaired two-tailed Student’s t-test; ***P < 0.001; n = 4 per group in the experiment). (**c**,**d**) WT B6 mice were i.v. injected with either PBS or GO (50 μg) and 14 hrs later total splenocytes were prepared from each group of mice. (**c**) FACS plots of DCs (CD11c^+^), macrophages (CD11c^−^CD11b^+^F4/80^+^), and iNKT cells (CD3ε^+^αGC/CD1d dimer^+^) in the spleen from each experimental group are shown. (**d**) The absolute numbers of these cells were measured by flow cytometry. (**e**) Intracellular TGFβ production in these cells was assessed by flow cytometry. The mean values ± SD are shown (unpaired two-tailed Student’s t-test; ***P < 0.001; n = 4 per group in the experiment). (**f,g**) WT and Jα18 KO B6 mice were i.p. injected with either PBS or GO (50 μg). Five days later, total splenocytes were prepared from PBS- or GO-injected mice. (**f**) The frequency of CD25^+^Foxp3^+^ Treg cells among total splenic CD4^+^ T cells from each group of mice was measured by flow cytometry. Representative data (left panels) and a summary (right panel) are shown. The mean values ± SD are shown (unpaired two-tailed Student’s t-test; **P < 0.01; n = 4 per group in the experiment). (**g**) Expression of GITR and FR4 in splenic CD4^+^CD25^+^Foxp3^+^ Treg cells from each experimental group was analyzed by flow cytometry. The mean values ± SD are shown (unpaired two-tailed Student’s t-test; **P < 0.01, ***P < 0.001; n = 4 per group in the experiment). Two-way ANOVA (genotype × treatment) showed a significant difference between these two factors (^##^P < 0.01 and ^###^P < 0.001).

The TGFβ1 signaling pathway plays a central role in GO-mediated anti-inflammatory responses, whereas the TLR4-NFκB axis has been implicated in regulating inflammatory immune responses elicited by a reduced form of GO (rGO)^24^. Since TGFβ promotes Treg cell differentiation, we asked whether GO can induce the expansion of Treg cells *in vivo*. For this purpose, we examined the frequency of Foxp3^+^ cells among CD4^+^ T cells after i.v. injection of GO. Interestingly, we found that the frequency of Treg cells was approximately 2-fold higher in GO-treated mice compared with vehicle-treated mice (Fig. 5a). In addition, the expression levels of Treg-related molecules such as GITR and FR4 on Treg cells were significantly elevated by GO (Fig. 5b).

Since Treg cell differentiation critically depends on TGFβ, we considered that GO might induce TGFβ expression in innate cells such as DCs, macrophages, and iNKT cells. The frequency and absolute cell number of innate cells (DCs, macrophages, and iNKT cells) were examined at 14 hrs after i.v. GO injection into WT B6 mice, but no differences were observed as compared with PBS injection (Fig. 5c,d). However, GO significantly increased TGFβ expression in iNKT cells but not DCs or macrophages (Fig. 5e). These data suggest that at early time points after GO stimulation, iNKT cells might serve as the main source of TGFβ, which is critical for initiation of Treg cell differentiation. To examine whether the GO-induced increase of Treg cells involves iNKT cells, we took advantage of Jα18 KO B6 mice. Upon *in vivo* GO stimulation, the frequency of Treg populations was significantly increased in WT B6 mice but remained largely unchanged in Jα18 KO B6 mice, suggesting that iNKT cells are the relevant cell type responsible for GO-induced expansion of Treg cells (Fig. 5f). In line with these findings, the expression levels of GITR and FR4 on Treg cells were significantly increased in GO-treated WT compared with Jα18 KO B6 mice (Fig. 5g).

Taken together, these results provide evidence that GO-induced phenotypic changes in iNKT cells can influence Treg cells *in vivo*.

## Discussion

Although previous studies have reported that GO interacts with APCs such as DCs and macrophages, and that GO downregulates CD1d expression on DCs^12,25^, its effects on iNKT cells have remained unexplored. Herein, we investigated whether GO can influence iNKT cell activities and found that it induces a phenotypic switch in these cells for production of the immunosuppressive cytokine TGFβ. Because mortality in certain models of septic shock has been attributed to iNKT cell activation, we hypothesized that *in vivo* GO treatment polarizes iNKT cells towards an anti-inflammatory phenotype, resulting in a dampened cytokine storm. Our results demonstrated that GO treatment attenuates inflammation in a model of iNKT cell-driven sepsis, via its capacity to polarize iNKT cell responses.

Unlike Gram-negative bacteria containing LPS, Gram-positive pathogens such as group B *Streptococci*, which cause neonatal sepsis and meningitis, contain diacylglycerol-containing glycolipids with the capacity to activate iNKT cells in a CD1d-dependent manner, ultimately leading to septic shock^2^. Moreover, severe liver injury, which is also seen during sepsis, can be caused by hepatic iNKT cells that are activated by intestinal microflora-derived glycolipids^26^. In addition to foreign glycolipid antigens, endogenous glycolipids such as β-D-glucopyranosylceramide (β-GlcCer) and isoglobotriaosylceramide (iGb3) accumulate in bacteria-stimulated APCs, resulting in iNKT cell-mediated inflammation^27,28^. These studies suggest both bacteria-derived and self-glycolipid antigens synthesized in bacteria-stimulated APCs as potential therapeutic targets of iNKT cell-mediated septic shock. Upon exposure to α-GalCer-mimicking glycolipid antigens derived from *S*. *pneumonia*, one of the causative pathogens of sepsis^27^, iNKT cells become activated and initiate inflammatory immune responses by secreting high levels of pro-inflammatory cytokines such as IFNγ, which subsequently activate other immune cells including NK and γδ T cells, ultimately amplifying inflammation^4,5^. Thus, our findings suggest that direct targeting of iNKT cells with GO could be effective in modulating early steps of inflammatory immune responses leading to glycolipid-mediated sepsis.

Prior studies have reported that GO triggers inflammatory responses by macrophages through TLR4-MyD88 signaling^29^ to induce necrotic cell death via autocrine TNFα stimulation^12^, indicating that TLR4 plays a key role in GO-mediated innate immune responses. However, another study showed that the TGFβ signaling pathway plays a critical role in triggering macrophage apoptosis induced by pristine graphene^30^. Based on the previous studies, GO can interact with two distinct receptors, TLR4 and the TGFβ receptor^24,30^. GO with high oxidant state more likely activates the TGFβ receptor signaling pathway leading to anti-inflammatory responses, whereas rGO with low surface oxidant state preferentially activates the TLR4 signaling pathway resulting in pro-inflammatory responses. Since these two signaling pathways act on each other in an antagonistic manner and because GO used in this study has a high oxidant state (it activates immune cells via TGFβ receptor rather than TLR4), blockade of TLR4 signaling pathway employing TLR4 mutant mice might eliminate any antagonistic effect of TLR4 signaling on TGFβ signaling, consequently resulting in enhancement of anti-inflammatory GO effects. Moreover, our findings indicate that the induction of TGFβ^+^ iNKT cells is closely related with suppression of α-GalCer-mediated immune responses, even though the TLR4 pathway exerts antagonistic effects on these responses.

Activation of innate immune cells (e.g. DCs and macrophages) by cytokines derived from α-GalCer-stimulated iNKT cells enhances adaptive immune responses^31^. Furthermore, iNKT cells enhance the generation of antigen-specific Th1 and Th2 responses *in vivo*^32^. Since GO modulates α-GalCer-mediated APC activation, we considered that GO might be able to influence antigen-specific CD4^+^ T cell polarization induced by α-GalCer. Our results demonstrated that GO injection significantly suppresses antigen-specific Th1, Th2, and Th17 polarization (Supplementary Fig. 2), which is consistent with previous studies showing that GO exposure attenuates Th2 immune responses in a model of OVA-induced asthma via suppressing OVA antigen-specific IgE and Th2 cells^13,33^. In addition, α-GalCer injection promotes a significant increase in OVA-specific Th2 polarization in an iNKT cell-dependent manner, suggesting that iNKT cell-derived IL4 contributes to the induction of allergic asthma. Moreover, some common allergens such as certain fungi and house dust extracts contain glycolipids that can activate iNKT cells to influence the pathogenesis of allergic responses^34,35^. Previous studies have suggested that, due to its tissue distribution and urinary excretion, the route of GO administration is a critical factor in its biological applications^36,37^. It has been reported that the tissue distribution of distinct iNKT subsets is highly variable: IFNγ-producing NKT1 cells and IL17-producing NKT17 cells predominate in the spleen and liver, whereas IL4-producing NKT2 cells are enriched in the lung and mesenteric lymph nodes (mLN). In line with this tissue distribution, i.v. injection of α-GalCer selectively activates NKT1 cells in the splenic red pulp and liver, whereas oral administration preferentially activates NKT2 cells in the mLN^38^. Therapeutic application of GO will need to take these findings into consideration when deciding on its injection route. Because GO can suppress iNKT cell-mediated immune responses, our findings identify GO as a promising therapeutic modality of iNKT cell-mediated allergic responses elicited by exposure of allergens containing glycolipid antigens.

It has been reported that the surface oxidant state of GO is linked with inhibition of LPS-induced IL6 production^39^. Additionally, TGFβ-mediated signaling plays a critical role in GO-mediated biological/toxicological effects on human hepatoma HepG2 cells, whereas rGO regulates immune responses via the TLR4-NFκB pathway^24^. Furthermore, GO has a negative surface charge mediated by an increase in acidic groups, including epoxy (C–O–C), hydroxyl (OH), and carboxyl (COOH) groups, on its backbone and edges, as compared with graphene. Such negative surface charge of GO might mediate suppressive effects on inflammatory responses mediated by LPS-stimulated macrophages, in a TGFβ-dependent mechanism^40,41^. Consistent with these previous studies, we observed that rGO, unlike anti-inflammatory GO, did not have any inhibitory effects on α-GalCer-mediated immune responses, indicating that GO and rGO induce distinct signaling responses (unpublished preliminary data). Thus, selective activation of TGFβ- rather than TLR4-mediated responses by GO used in this study can be explained by its chemical properties such as negative surface charge due to high oxygen contents.

Taken together, we have demonstrated that GO is a key regulator of iNKT cell activation and that it can modify the phenotype of iNKT cells towards a tolerogenic one. The following is the most likely scenario by which GO can suppress α-GalCer-triggered iNKT cell activation in an APC-dependent manner: GO can directly induce phenotypic changes in iNKT cells by converting these cells from an IFNγ-producing inflammatory phenotype to a TGFβ-producing regulatory phenotype. Such GO-stimulated iNKT cells therefore increase TGFβ production but decrease IFNγ production even after recognition of glycolipid antigen (α-GalCer) loaded on CD1d expressed on APCs such as DCs and macrophages. This, in turn, suppresses α-GalCer-induced inflammatory cascades mediated by various innate immune cells including DCs, macrophages, NK cells, and γδ T cells. Thus, the regulatory effects of GO on iNKT cell-mediated innate immune responses contribute to the protection against iNKT cell-induced pathogenesis during septic shock. In conclusion, our findings provide a promising and testable strategy to design GO as a nano-platform with intrinsic immune modulatory properties to deliver future sepsis drugs.

## Methods

### Mice and reagents

Wild-type (WT) C57BL/6 (B6), TLR4-mutant (C3H/HeJ), and TLR4-WT (C3H/HeN) mice were purchased from Central Laboratory Animal, Inc. (Seoul, Korea). Jα18 KO B6 mice were kindly gifted by Dr. M. Taniguchi (RIKEN, Yokohama, Japan) and invariant Vα14 TCR transgenic (Tg) B6 mice were kindly provided by Dr. A. Bendelac (University of Chicago, Chicago, IL, USA). DO11.10 OVA-specific TCR Tg mice with a Balb/c background were used in this study. All mice were used at the age of 6–12 weeks for experiments, and maintained at Sejong University. They were maintained on a 12-hour light/12-hour dark cycle in a temperature-controlled barrier facility with free access to food and water. These mice were provided with autoclaved tap water and fed a γ-irradiated sterile diet. In this study, gender- and age-matched mice were used for all experiments. The animal experiments were approved by the Institutional Animal Care and Use Committee (IACUC) at Sejong University (SJ-20161103), and all experiments were performed in accordance with the relevant guidelines and regulations. α-GalCer (KRN7000) was purchased from Enzo Life Sciences, Inc. (Farmingdale, NY, USA). Nano graphene oxide (NANO-GO-P) was purchased from Graphene Supermarket (Calverton, New York, USA) and was dispersed in distilled water as the stock solution (10 mg/ml).

### Measurements and characterization of GO

Elemental analysis (C, H, N, S) was carried out on a Flash EA-2000 Elemental Analyzer (Thermo Finnigan, Milano, Italy) by employing tungstic anhydride and copper wire as catalysts. The element contents were calculated for oxygen by an element analyzer, a Flash EA-1112 (Thermo Scientific, The Netherlands) with nickel plated carbon and quartz turnings as catalysts. Raman spectroscopy was recorded by using Micro-Raman spectrophotometer (Nanofinder FLEX G, Lambda Ray) at a wavelength of 532 nm. Morphology of GO were observed by high resolution transmission electron microscopy (TEM) (Talos F200X microscope operating at 200 kV, FEI). Specimens for TEM imaging were prepared by sprinkling a drop of sample on copper grids and evaporating in vacuum.

### Induction of septic shock

For induction of septic shock, mice were i.p. injected with α-GalCer (2 µg/mouse) plus 2-amino-2deoxy-D-galactose (D-galactosamine) (25 mg/mouse) after intravenous (i.v.) injection of either vehicle or GO. All animals were continuously monitored for α-GalCer/D-GalN-induced lethality for 72 hrs after challenge.

### Flow cytometry

The following monoclonal antibodies (mAbs) were obtained from BD Biosciences (San Jose, USA): allophycocyanin (APC)-, or phycoerythrin (PE)-conjugated anti-NK1.1 (clone PK-B6); APC-, or PE-Cy7-conjugated anti-CD11c (clone HL3); PE-Cy7-conjugated anti-GITR (clone DTA-1); PE-conjugated anti-MHC II (clone M5/114.15.2); APC-, or PE-Cy7-conjugated anti-CD3ε (clone 145-2C11); PE-conjugated anti-CD1d (clone 1B1); PE-Cy7-conjugated anti-CD11b (clone M1/70); PE-conjugated anti-CD40 (clone 3/23); PE-Cy7-conjugated anti-CD69 (clone H1.2.F3); PE-conjugated anti-IL12p40 (clone C15.6); biotin-conjugated anti-CD86 (clone GL1); APC-conjugated anti-F4/80 (clone BM8); PE-conjugated anti-TNFα (clone XP6-XT22); PE-conjugated anti-IL6 (clone MP5-20F3); PE-conjugated anti-IL4 (clone BVD6-24G2); APC-, or PE-Cy7-conjugated anti-CD4 (clone RM4-5); and PE-conjugated anti-IgG1 (κ isotype control). The following mAbs from eBioscience were used: fluorescein isothiocyanate (FITC)-conjugated anti-KJ1-26 (clone KJ1-26); PE-conjugated anti-IL17A (clone eBio17B7); PE- or FITC- conjugated anti-Foxp3 (clone NRRF-30); PE-conjugated anti-TGFβ1/LAP (clone TW7-16B4); PE-conjugated anti-FR4 (clone eBio12A5); and PE-conjugated anti-IFNγ (clone XMG1.2). The acquisition of flow cytometric data were collected using a FACSCalibur ^TM^ flow cytometer (Becton Dickinson, USA) and analyzed using FlowJo software (Tree Star, USA). To perform surface staining, cells were harvested, washed twice with cold 0.5% BSA-containing PBS (FACS buffer) and then were incubated with anti-CD16/CD32 mAbs on ice for 10 min for blocking Fc receptors. Subsequently, these cells were stained with fluorescence-labeled mAbs. Flow cytometric data were acquired using a FACSCalibur flow cytometer (Becton Dickson, San Jose, CA, USA) and analyzed using FlowJo software (Tree Star Inc., Ashland, OR, USA).

To perform surface staining, cells were harvested, washed twice with cold 0.5% BSA-containing PBS (FACS buffer) and then were incubated with anti-CD16/CD32 mAbs on ice for 10 min for blocking Fc receptors. Subsequently, these cells were stained with fluorescence-labeled mAbs. Flow cytometric data were acquired using a FACSCalibur flow cytometer (Becton Dickson, San Jose, CA, USA) and analyzed using FlowJo software (Tree Star Inc., Ashland, OR, USA).

### CD1d/α-GalCer dimer staining for iNKT cells

To stain iNKT cells specifically, mCD1d/Ig fusion proteins (CD1d dimer; mouse CD1d dimerX, BD Biosciences) were incubated overnight at 37 °C with 40-fold molar excess of α-GalCer (in PBS containing 0.5% Tween 20). The staining cocktail was prepared by mixing α-GalCer-loaded mCD1d/Ig proteins with FITC- or APC-conjugated anti-mouse IgG1 Ab (clone A85-1, BD PharMingen, San Diego, CA) at a 1:2 ratio of dimer to anti-mouse IgG1 Ab. Subsequently, the mixture was incubated for 2 hrs at room temperature (RT).

### Intracellular cytokine staining

To perform intracellular staining, single-cell suspensions from the spleen were incubated with 10 μg/ml intracellular protein transport inhibitor (brefeldin A), in RPMI medium for 2 hrs at 37 °C. The cells were stained for cell-specific surface markers, then fixed with 4% paraformaldehyde in PBS, washed with cold (4 °C) FACS buffer, and permeabilized with permeabilization solution (1% BSA and 5% saponin in PBS) for 10 min. Subsequently, the permeabilized cells were stained for 30 min at RT with the indicated anti-cytokine mAbs (PE-conjugated anti-IL12p40, anti-IL4, anti-TNFα, anti-IL6, anti-IFNγ, anti-IL17A, and anti-TGFβ1; PE-conjugated rat IgG isotype control mAbs). Intracellular staining for transcription factor was performed using the Foxp3/transcription factor staining buffer set kit (eBioscience, USA) with the indicated mAbs (PE- or FITC-conjugated anti-Foxp3; or PE-conjugated rat IgG isotype control mAbs). Samples (more than 5,000 cells) were acquired using a BD FACSCalibur and analyzed with the Tree Star FlowJo software package.

### Isolation of liver leukocytes

Mice were anesthetized using a combination of ketamine and xylazine at 40 mg/kg and 4 mg/kg, respectively, and were perfused via the left heart ventricle with cold sterile PBS for 3 min to remove PBMCs from the blood vessels. The liver was removed after perfusion, cut into small pieces by scissors and a scalpel and digested with collagenase type IV (Sigma, St. Louis, MO, USA; 2.5 mg/ml) and DNase I (Promega, Madison, USA; 1 mg/ml) for 15 min at 37 °C. Subsequently, the digested tissues were dissociated into single-cell suspensions using combination C Tubes and gentleMACS^TM^ dissociator (Miltenyi, Germany). The digested tissues were filtered using a 70-μm-pore cell strainer, and subsequently the cells were washed once with PBS (10% FBS). Mononuclear cells were collected from the 40/70% Percoll interphase after discontinuous Percoll gradient. The number of total mononuclear cells was determined using 0.4% trypan blue (Welgene, Seoul, Korea) and a hemocytometer before staining after washing with PBS.

### Cell isolation by magnetic activated cell sorting (MACS)

A single-cell suspension from the spleen was harvested and resuspended in RPMI complete medium consisting of RPMI 1640 (Gibco BRL, USA) medium supplemented with 10 mM HEPES, 2 mM L-glutamine, 10% FBS, 5 mM 2-mercaptoethanol, and 100 units/mL penicillin-streptomycin. iNKT cells were enriched using NK1.1^+^ iNKT cell isolation kit (Miltenyi, Germany). The enriched iNKT cell population was >88% pure after MACS. CD11c^+^ DCs and CD4^+^ T cells were isolated from mice using a MACS system (Miltenyi, Germany). CD11c^+^ DC population was >94% pure after MACS and the CD4^+^ T cell population was >94% pure.

### *In vivo* T helper cell differentiation assay

DO11.10 TCR Tg Balb/c mice were immunized i.p. with α-GalCer (2 µg) plus OVA_323–339_ peptide (100 µg) after i.v. injection of either GO or vehicle. Five days later, CD4^+^ T cells were isolated from the immunized mice and then co-cultured with DCs pulsed with OVA_323–339_ (10 μg/ml) at a ratio of 10 to 1 for 24 hrs. Cytokine production was determined by intracellular cytokine staining.

### Statistical analysis

Two-way ANOVA analysis was performed using VassarStats (http://faculty.vassar.edu/lowry/VassarStats.html). ^#^P < 0.05, ^##^P < 0.01, and ^###^P < 0.001 indicate significant differences in the two-way ANOVA. Unpaired two-tailed Student’s t-test was performed for the comparison of two groups using Excel (Microsoft, USA). *P < 0.05, **P < 0.01, and ***P < 0.001 are considered significantly different in the unpaired two-tailed Student’s t-test.

## Electronic supplementary material

Supplementary Information
