# Supplementary material for: Graphene oxide polarizes iNKT cells for production of TGFβ and attenuates inflammation in an iNKT cell-mediated sepsis model
Source: Sci Rep. 2018 Jul 4;8:10081. doi: 10.1038/s41598-018-28396-9 (PMC6031608; doi:10.1038/s41598-018-28396-9)
Supplement: Supplementary file 1 — Supplementary Information [file 41598_2018_28396_MOESM1_ESM.docx]

**Supplementary Information**

**Graphene oxide polarizes iNKT cells for production of TGFβ and attenuates inflammation in an iNKT cell-mediated sepsis model**

Sung Won Lee ^1, 2¶^, Hyun Jung Park ^1¶^, Luc Van Kaer ^3^, Suklyun Hong ^2, 4^*, and Seokmann Hong ^1,^*

^1^Department of Integrative Bioscience and Biotechnology, Institute of Anticancer Medicine Development, Sejong University, Seoul 05006, Korea

^2^Graphene Research Institute, Sejong University, Seoul 05006, Korea

^3^Department of Pathology, Microbiology and Immunology, Vanderbilt University School of Medicine, Nashville, TN 37232, USA

^4^Department of Physics, Sejong University, Seoul 05006, Korea

*Corresponding authors: Tel: 82-2-3408-3649; Fax: 82-2-466-4187; E-mail: [shong@sejong.ac.kr](mailto:shong@sejong.ac.kr); Tel: 82-2-3408-3209; Fax: 82-2-497-2634; E-mail: hong@sejong.ac.kr

^¶^These authors contributed equally to this work.

**Running title:** Attenuation of a-GalCer/D-Gal-induced sepsis by GO-polarized iNKT cells

**Keywords:** Graphene oxide, 𝛼-Galactosylceramide, iNKT cells, TLR4, TGFβ, septic shock

**
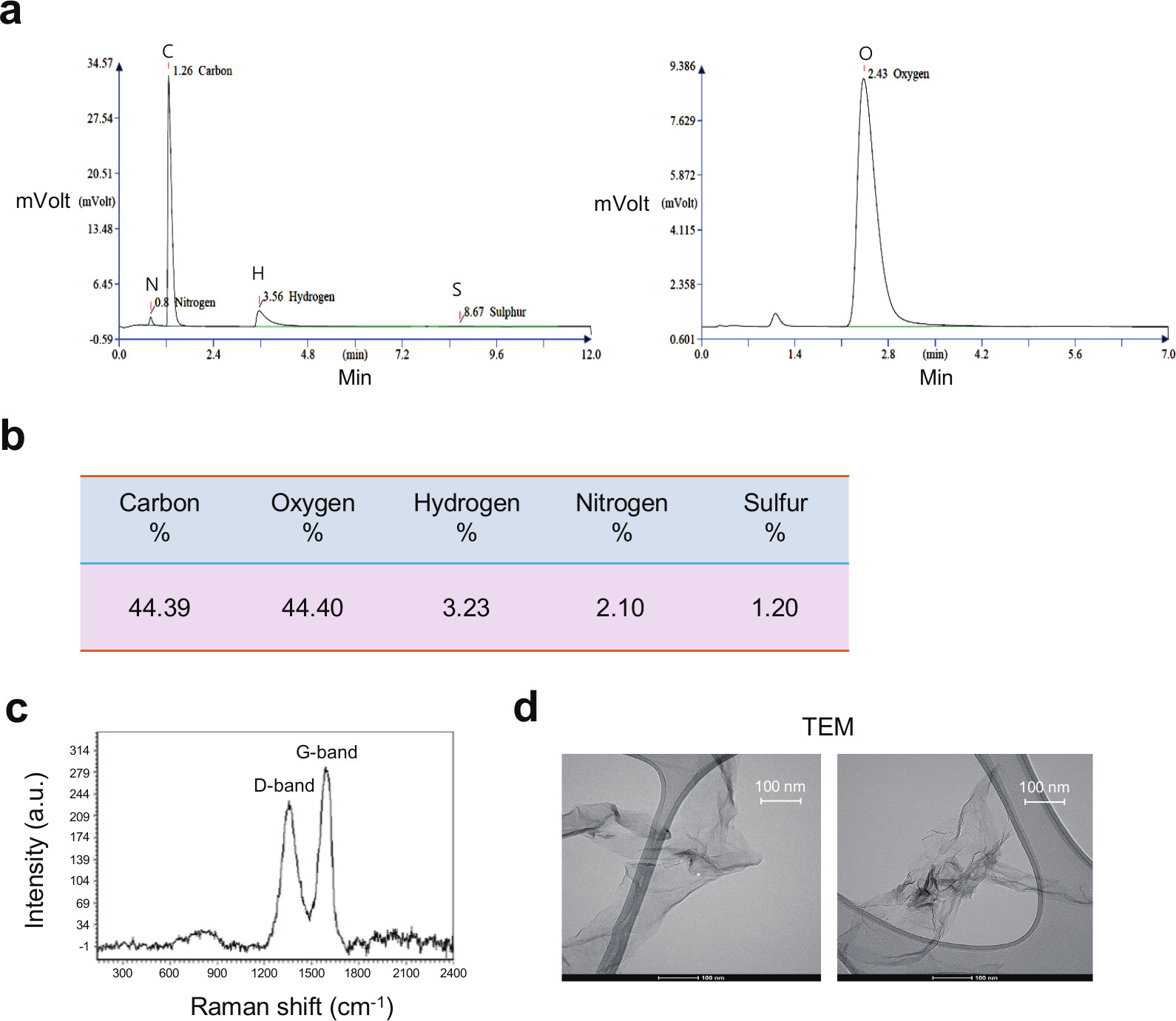
**

**Supplementary Figure 1. Characterization of GO used in this study.**

(a-b) Elemental composition of GO determined by elemental analysis. (a) EA raw data. (b) The percentages of carbon, oxygen, hydrogen, nitrogen, and sulfur in GO. The major elements of an organic substance, namely, carbon, hydrogen, and nitrogen, are commonly determined using elemental analyzers. Our data showed that both oxygen and carbon content of GO was similar (44 wt%), indicating that the ratio of oxygen/carbon was about 1.0. (c) Raman spectrum of GO was measured using Raman spectrophotometer. Raman spectrum was used to obtain structural information about GO. The spectrum of graphite shows a strong G band at 1570cm^-1^ arising from the first-order scattering of the E2g phonon of sp2 carbon atoms, whereas GO increased intensity of the D band at 1350cm^-1^ which related to the size of the in-plane sp2 domains in addition to G band. The increase of the D peak intensity in Raman spectra of GO indicates forming more sp2 domains (d) TEM images of GO. The analysis of morphology was performed by TEM. As expected, GO is smaller than one micrometer in diameter.

**
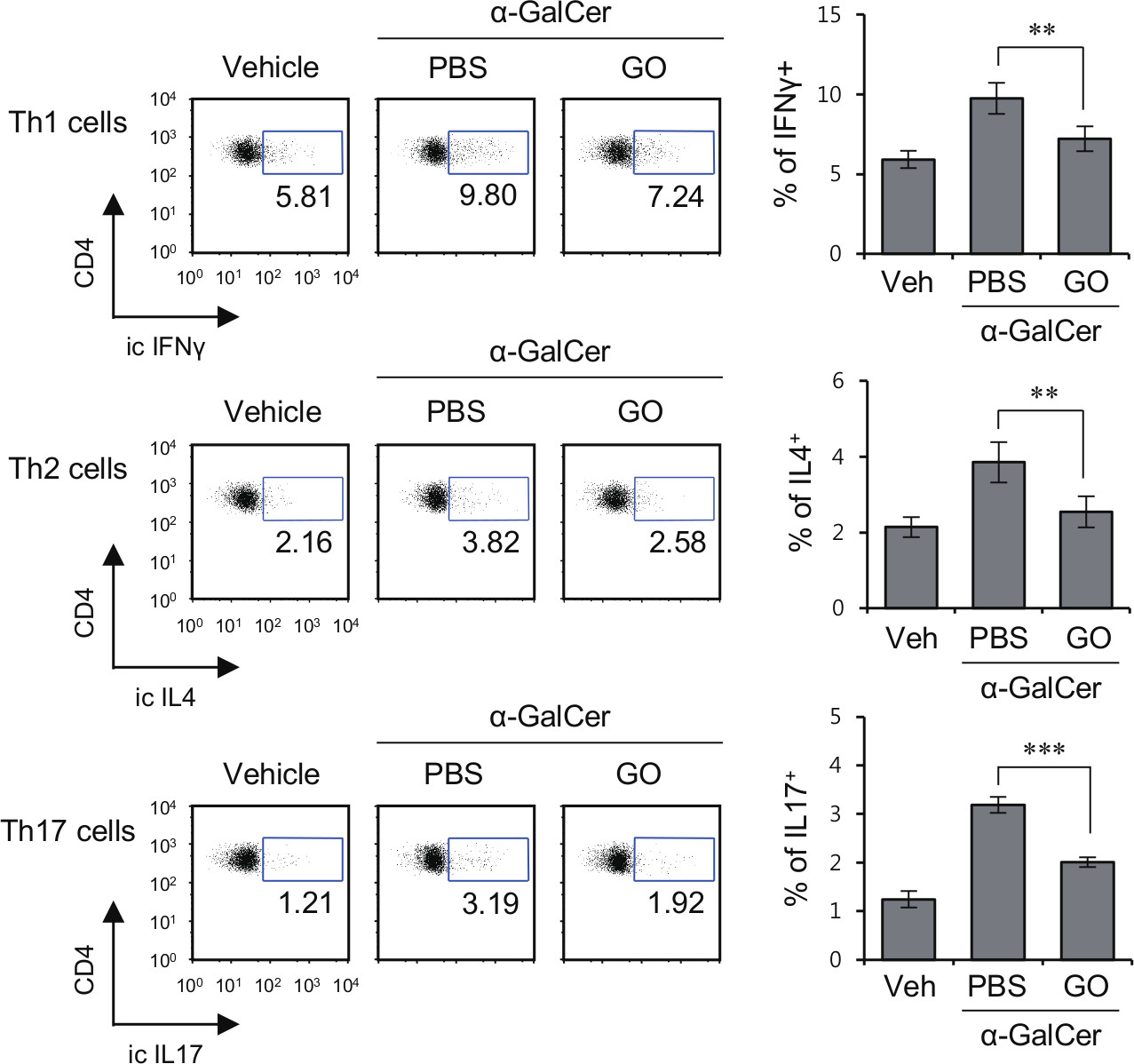
**

**Supplementary Figure 2. GO inhibits α-GalCer-mediated adjuvant effects on adaptive immunity.**

DO11.10 TCR Tg mice were injected with either GO (50 μg) or GO + α-GalCer (2 μg). After 5 days, splenic CD4^+^ T cells were purified from mice and were subsequently restimulated *in vitro* for 24 hrs with OVA-pulsed DCs. Intracellular IFNγ, IL4, and IL17 production in CD4^+^ T cells (KJ1-26^+^CD3ε^+^CD4^+^) was assessed via flow cytometry. The mean values ± SD are shown (n = 4 per group in the experiment; unpaired two-tailed Student’s t-test; **P<0.01, ***P<0.001).
